# Atrial fibrosis in atrial fibrillation pre-ablation assessed by CMR: impact in atrial size and function?

**DOI:** 10.1186/1532-429X-13-S1-P259

**Published:** 2011-02-02

**Authors:** Ana G Almeida, Joao Sousa, Luis Carpinteiro, Joao S Marques, Nuno Cortez-Dias, Claudio David, Claudia Jorge, Doroteia Silva, Mario G Lopes, Antonio N Diogo

**Affiliations:** 1 University Hospital Santa Maria, Lisbon, Portugal

## Introduction

Atrial fibrillation (AF) is associated with different amounts of diffuse fibrosis, which has impact in the therapy response. CMR is able to assess myocardial fibrosis using late gadolinium enhancement (LGE) and chamber function.

## Purpose

The aim of this study was to assess, in patients with AF if the presence and extension of atrial fibrosis was related with atrial size and function.

## Methods

47 (42 male, 42±8 years-old) consecutive patients (pts) with paroxysmal AF and indication to ablation were included. Exclusion criteria for CMR: non-sinus rhythm and general contra-indications. Before ablation all underwent CMR for left atrial angiography for CARTO mapping. Using CMR, the following variables were assessed: left ventricle (LV) and left atrium (LA) volumes and ejection fraction (SSFP, short-axis), LV LGE was assessed as presence and amount (LGE mass/LV mass). LA LGE location was assigned to 3 atrial walls: anterior, posterior and septal. The extension was classified in 3 degrees, according to the number of atrial walls with LGE: 1=one wall, 2=two walls; 3=three walls. For CMR analysis dedicated software was used (Philips View Forum Release 6.1).

## Results

All studies yielded good image quality. LV end-diastolic volume was 68±5ml/m2 and normal in all; LV ejection fraction was 58±4%(48-67), normal in all (in 2 borderline); 11 pts showed minimal LV LGE involving one or two segments with 0.02% of fibrosis mass/LV mass; mean LA volume was 115±21ml (increased in 45% o patients); mean LA ejection fraction was 45±11% (reduced in 65%). Type 1 LGE was found in 17 pts, type 2 in 22 and type 3 in 8 pts. Comparing the groups with the 3 LGE types, pts with type 3 LGE had larger LA volumes and lower ejection fraction in comparison with type 1 LGE pts (p=0.004 and p=0.001 respectively). No difference was found between other groups. No relationship was found with LV volumes, ejection fraction and amount of fibrosis.

## Conclusion

In pts with paroxysmal AF pre-ablation, atrial fibrosis as assessed semi-quantitatively by CMR was associated to larger left atrial volume and lower ejection fraction, which are determinant substrates for the outcome.

